# Residents’ Disaster Preparedness after the Meinong Taiwan Earthquake: A Test of Protection Motivation Theory

**DOI:** 10.3390/ijerph15071434

**Published:** 2018-07-07

**Authors:** Jing-Shia Tang, Jui-Ying Feng

**Affiliations:** 1 Department of Nursing, Chung Hwa University of Medical Technology, No. 89, Wenhua 1st St., Rende Dist., Tainan 71703, Taiwan; tangjeanine@mail.hwai.edu.tw; 2 International Doctoral Program in Nursing, College of Medicine, National Cheng Kung University, No. 1, University Rd., Tainan 70101, Taiwan; 3 Department of Nursing and Institute of Allied Health Sciences, College of Medicine, National Cheng Kung University, Tainan 70101, Taiwan; 4 Department of Nursing, National Cheng Kung University Hospital, No. 1, University Rd., Tainan 70101, Taiwan

**Keywords:** disaster preparedness, protection motivation theory, earthquake, Taiwan

## Abstract

Because effective preparations are required to mitigate disaster impacts before implementing effective interventions, it is important to understand why people do or do not act on disaster preparedness. This study explores factors influencing residents’ intentions and actual behaviors following the 2016 Kaohsiung Meinong earthquake in southern Taiwan. Protection Motivation Theory was used to develop a hypothesized model to test hypotheses regarding residents’ disaster preparedness, and structural equation modeling (SEM) was used to test the model. Data were comprised of 286 valid responses from seven major administration areas in Tainan, Taiwan. Self-efficacy, response-efficacy, and obstacles were significantly correlated with behavioral intentions and actual disaster preparedness behaviors. SEM results revealed that (a) the model fit the data well, (b) the relationship between risk perception and response-efficacy was fully mediated by behavioral intention, and (c) self-efficacy and obstacles were partially mediated by behavioral intention. Behavioral intent and actual disaster preparedness behavior are related but not equal. The main factors affecting actual disaster preparedness behavior are self-efficacy and obstacles. Therefore, strategies like drills or workshops can improve disaster-preparedness knowledge and capabilities and reduce difficulties of implementing disaster preparedness. To improve health and well-being, healthcare providers should promote disaster preparedness by interventions to increase self-efficacy during disasters.

## 1. Introduction

Only effective disaster preparedness can mitigate the impacts future disasters are likely to have on human lives, health, and property [1,2]. Enhancing preparedness for hazardous events is mainly concerned with mitigating damage and addressing potential threats [3,4]. Earthquakes are among the most powerful natural forces in the world and differ from other destructive phenomena such as typhoons, tornadoes, and floods that are precipitated by extreme climates. Although earthquakes occur less frequently than other natural disasters, because of their unpredictability, people need to prepare for them before they occur.

Taiwan is located in an active seismic region, and the epicenter of the Meinong earthquake occurred in Kaohsiung City, Taiwan, at 3:57 a.m. on 6 February 2016. The depth of the source that generated these seismic waves was 14.6 km, and the event measured 6.6 on the Richter magnitude scale. The earthquake caused the deaths of 117 people and injured 510 people in Tainan City, which is about 50 km from Kaohsiung City. Eight houses were completely destroyed, and five houses partially collapsed [5]. The participants in this study are residents who experienced this earthquake. Some had taken preparedness actions, while others had not.

Preparedness is not only a form of disaster management, but also a dynamic form of health promotion [6]. Therefore, healthcare providers have a responsibility to promote disaster preparedness among individuals, households, and communities [7] to improve people’s health and well-being. To increase disaster preparedness behavior among residents, it is necessary to explore factors related to disaster preparedness motivations and behaviors prior to the implementation of appropriate disaster preparedness strategies.

Protection Motivation Theory (PMT)—a health promotion model—states that some form of risk information can provide the impetus for an individual to determine the degree of risk severity, their vulnerability, and their ability to reduce that risk. PMT was first proposed by Rogers [8] in 1975 (and revised in 1985) to describe the mechanisms people use to adopt protective behaviors and reduce perceived threats. It explains that a cognitive process informs the efforts taken to protect oneself from harm and can be used to analyze both maladaptive behavior and adaptive response. For example, maladaptive responses are those that place an individual at health risk by either engaging in a health behavior risk (e.g., smoking) or not adopting a protective behavior or adaptive responses (e.g., getting vaccinated). PMT posits that health protection behaviors and disaster preparedness intentions are motivated by the same principles. Therefore, Grothmann and Patt [9] suggest that PMT can be used to explore disaster preparedness behavior. Thus, PMT has become one of the most widely applied disaster prevention decision-making models [10]. Related studies have explored threat appraisals among people that considered (1) the severity of the threat (individuals’ estimates of likely harmful consequences and severity) and (2) the probability that the threat would come to pass (the probability that individuals would incur damage from a particular threat). When people appraise a threat, they come to realize the existence of certain risks. Therefore, threat appraisal is also referred to as risk perception [11]. Self-efficacy and response-efficacy are essential components of an effective coping strategy [12] and refer to the existing levels of individuals’ abilities in relation to the recommended level of disaster preparedness and its effectiveness [13]. Furthermore, behavioral intentions may be influenced by both an individuals’ appraisal of a threat and their ability to cope.

How people respond to disasters depends on their assessment of the damage likely to be inflicted on their environment and them. Similar to preventive medicine, one preventive measure that can be taken to mitigate the damage inflicted by a disaster includes improving people’s disaster preparedness. Although many people are fearful of the severe damage a natural disaster can cause them, their families, and society, they lack the motivation needed to engage in disaster preparedness [14]. In Taiwan, earthquake preparedness is mostly considered from the perspective of building structures [15,16,17,18] and is limited to considering factors that may influence residents to initiate behaviors that could protect them in the event of a disaster. Thus, when their disaster preparedness includes implementing effective prevention and mitigation measures, people develop abilities that will enable them to cope with various situations.

People’s attitudes towards disaster preparedness are influenced by their personal characteristics, family circumstances [19,20], belief-related variables such as hazard beliefs, and how they assess their self-efficacy and outcome efficacy [13]. Lindell et al. [19] found that people with higher levels of disaster preparedness include families with vulnerable family members (e.g., elderly, children, and the disabled). Previous studies also found that other predictors of disaster preparedness behavior include high educational levels [13], home ownership, length of residence at their current address [21], perceived vulnerability [22], and previous disaster exposure [23,24]. In particular, perceived efficacy plays a critical role [25,26] in predicting disaster preparedness. Inconsistent results have also been found with regard to people’s risk perceptions. For example, Lindell and Perry [27] analyzed 23 studies and concluded that higher risk perceptions tended to result in actual disaster preparedness behaviors. Some studies, however, do not support the proposition that higher risk perceptions result in actual preparations being made [13,28]. A study of 885 residents living in France, for example, found that higher perceptions of risk positively influenced people’s intentions to implement preventive measures but did not significantly influence their preparedness [29].

**Figure 1:**
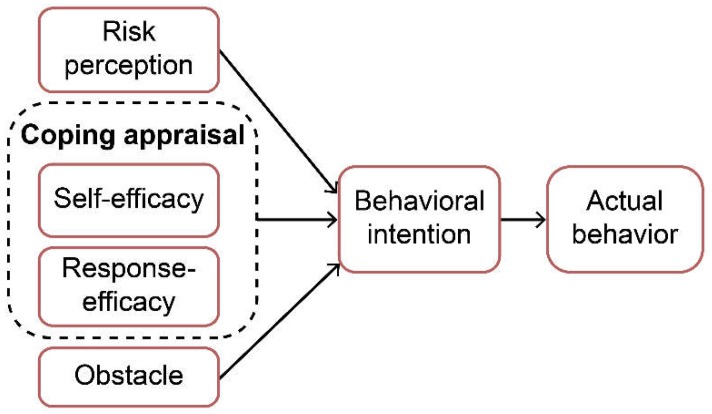
Behavioral intention factors affecting actual disaster preparedness behavior (Modified from the Protection Motivation Theory [12]).

Research on the social cognitive perspective that a person’s disaster preparedness behavior can be explained is limited in Taiwan, and the purpose of this study is to address this gap. It analyzes the levels of disaster preparedness that were evident following the earthquake in southern Taiwan in 2016, and it draws on a modified PMT to explore what factors motivated some residents to take steps to prepare themselves for a disaster. We also test that the obstacles can directly motivate or restrain intention and preparedness actions. We propose a path model of preparedness (as shown in Figure 1) that includes four domains (risk perception, self-efficacy, response-efficacy, and obstacles) as independent variables and one latent variable (behavioral intention) as a mediator of how disaster preparedness influenced actual behavior. This model hypothesizes that the effects of the four domains on preparedness actions may actually be indirect effects mediated by behavioral intentions.

Premised on the modified PMT, specific hypotheses were formulated and tested. Three of these hypotheses were as follows:

**Hypothesis** **1** **(H1).** *Disaster preparedness in terms of risk perception, self-efficacy, response-efficacy, and obstacles is dependent on personal characteristics.*

**Hypothesis** **1** **(H2).** *Residents’ risk perceptions, self-efficacy, response-efficacy, and obstacles can be associated with behavioral intentions and actual behavior in regard to disaster preparedness*.

**Hypothesis** **1** **(H3).** *Behavioral intention mediates the relationship between risk perception, self-efficacy, response-efficacy, obstacles, and actual behavior with regard to disaster preparedness*.

## 2. Materials and Methods

### 2.1. Design and Participants

This study was based on a two-step, descriptive, cross-sectional design. The first step involved conducting a preliminary study to develop a questionnaire and recruiting 30 Tainan residents as participants for a test-retest. During the second step, which represented the main part of the study, data were obtained from seven administrative areas in Tainan.

Participants had to be between 20 and 80 years of age, registered and living in Tainan City, have no mental illness, and be able to read and write Mandarin. Residents who were registered but did not live in Tainan or were not in Tainan during the Meinong Taiwan earthquake were excluded. The participants’ average age was 38.1 ± 10.95 years, with a range between 20 and 79 years. Most were women (65.7%) and had a college degree or higher (75.9%). Married and single persons each accounted for nearly half of all participants (49.3% vs. 48.3%, respectively). Those who lived with elderly family members accounted for 27.6% of the participants, and people with children under 18 years old accounted for 43.0% of the participants. Most had a religious affiliation, a job, and owned a house (Table 1).

According to Hoyle [30] and Loehlin [31], because structural equation modeling (SEM) relies on tests that are sensitive to sample size, the sample size should be at least 100, preferably 200. A total of 305 responses were retrieved. However, nine responses were invalid due to missing data and thus were excluded from subsequent analyses. The response rate was 94%, and 286 valid responses were analyzed. All participants had been living in their administrative areas from the day of the Meinong Taiwan earthquake in 2016 until the survey was conducted between October 2016 and July 2017.

### 2.2. Questionnaire

A self-developed questionnaire was designed based on the modified PMT (Figure 1). The self-reported questionnaire included 88 items that were used to measure disaster preparedness in relation to six subscales: risk perception, self-efficacy, response-efficacy, obstacle, behavioral intention, and actual behavior. Specific assessments were also made with regard to whether they had a stock of emergency supplies and had completed a family emergency plan and a home safety assessment. Before being finalized, the disaster preparedness questionnaire (DPQ) was reviewed and evaluated by five experts (the chief executive officer of the Emergency Operations Center, an assistant professor of public health, a lecturer of psychiatry nursing, an emergency room nurse, and a structural engineer), and four items were deleted because they had a lower mean score. The final questionnaire comprised 84 items. Respondents were asked to rank each item on a 5-point Likert scale from 1 (“strongly disagree” or “not serious at all,” depending on the question) to 5 (“strongly agree” or “very serious”). Participants were expected to be able to complete the questionnaire in 20 minutes. The psychometric properties of the DPQ were acceptable, with a content validity index of 0.95, Cronbach’s alphas from 0.78–0.95, and test-retest reliabilities of 0.44–0.79.

### 2.3. Data Analysis

Data analysis was conducted using SPSS 22.0 (SPSS Inc., Chicago, IL, USA), and the significance level was set at α < 0.05. *t*-tests were used to test for significant differences between the groups. Correlations between major variables were calculated. SEM with AMOS 21.0 (SPSS Inc., Chicago, IL, USA) was used to test the hypothesized model. The SEM was applied to assess the determinant factors for actual disaster preparedness behavior (ADPB). We used the four factors of PMT (risk perception, self-efficacy, response-efficacy, and obstacles) to predict ADPB. Subsequently, SEM analyses were performed using risk perception, self-efficacy, response-efficacy, and obstacles as the independent variables and ADPB as the dependent variable. The model evaluation criteria used to test the model’s fit were the ratio of the chi-square value to degrees of freedom (χ^2^/df), goodness of fit index (GFI), adjusted goodness of fit index (AGFI), comparative fit index (CFI), incremental fit index (IFI), non-normed fit index (NNFI), and root mean square error of approximation (RMSEA). The χ^2^/df should be no greater than 3 [32], GFI should be greater than 0.8 [33], and an AGFI value greater than 0.8 is acceptable [34]. The CFI and NNFI values should be greater than 0.90 [35]. IFI values over 0.9 indicate a good fit, but the index can exceed 1 [36]. The RMSEA value of 0.06 or less is indicative of an acceptable model fit, and values in the vicinity of 0.08 indicate a fair fit [37,38]. In addition, as suggested by Preacher and Hayes [39], the bias corrected bootstrapping method was applied in testing the mediating effects. Bootstrapping is a statistical technique that is widely valued for its capacity to effectively increase analytical power and control for Type I errors, especially when multivariate normality cannot be assumed in small samples [39,40].

### 2.4. Ethics Approval

The Ethics Review Board of the Jianan Psychiatric Center approved this study (No. 16-012), and all participants provided informed consent.

## 3. Results

### 3.1. Demographic Differences among Major Variables

Independent *t*-tests were used to analyze the characteristics of the participants in different groups. Men displayed lower scores in perceived risk (*t* = 2.671, *p* = 0.008) and behavioral intention (*t* = 2.399, *p* = 0.018) than women. The educational levels of participants with less than a college degree had a lower response-efficacy (*t* = −2.356, *p* = 0.019) and more obstacles (*t* = 3.299, *p* = 0.001) than those with college degree or higher. People with jobs had higher response-efficacy than those without jobs (*t* = 2.291, *p* = 0.023). There were no differences associated with religious affiliation, living with family members (over 65 years or under 18 years), and being homeowners.

### 3.2. Mean Scores and Correlations among Various Measures

Table 2 presents the mean scores and Pearson correlation coefficients of the key variables of interest. The mean scores of risk perception, self-efficacy, response-efficacy, and behavioral intentions in terms of disaster preparedness were higher than those of obstacles and actual behaviors. With the exception of risk perception, all of the other predictors—risk perception, self-efficacy, response-efficacy, and obstacles—were correlated with significant behavioral intentions and with actual disaster preparedness behaviors. The significantly correlated predictors were more highly correlated to behavioral intentions than actual disaster preparedness behaviors.

### 3.3. Structural Equation Modeling Analysis

**Figure 2:**
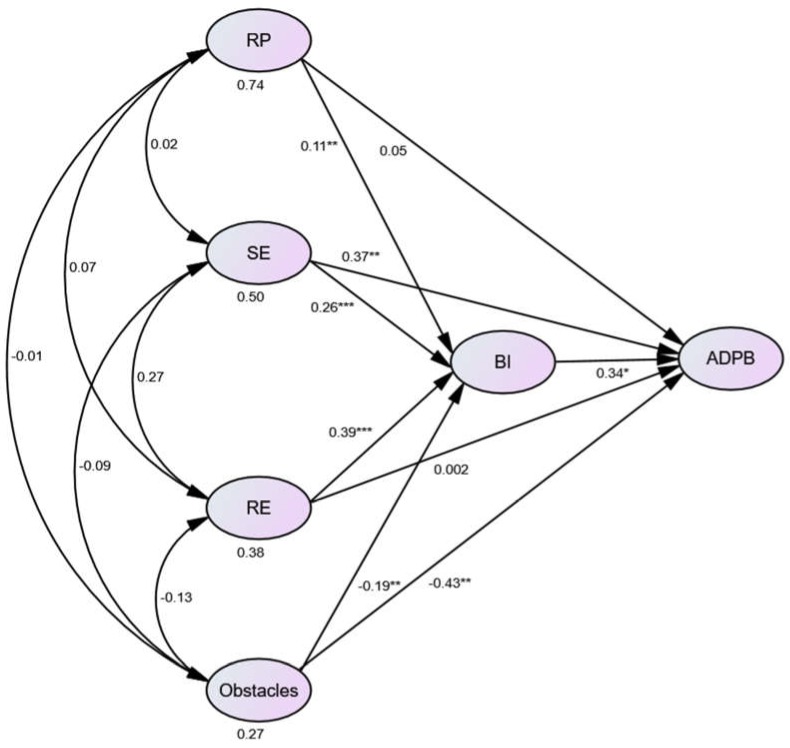
Structural equation modeling of the hypothesized model. * *p* ≤ 0.05. ** *p* ≤ 0.01. *** *p* ≤ 0.001; RP = risk perception; SE = self-efficacy; RE = response efficacy; BI = behavioral intention; ADPB = actual disaster preparedness behaviors.

The results of the structural equation modeling indicate that the hypothesized model fit the data well (*χ*^2^ = 741.905, df = 362, *χ*^2^/df = 2.049, GFI = 0.842, AGFI = 0.810, CFI = 0.918, IFI = 0.919, NNFI = 0.908, RMSEA = 0.061). A comparison of these results with the corresponding critical values suggests that the conceptual model fit the empirical data reasonably well [41]. Figure 2 shows the estimated model with unstandardized path coefficients.

We investigated the indirect effects of the dependent variable through the mediators, and direct and total effects; we performed percentile bootstrapping and bias-corrected percentile bootstrapping at a 95% confidence interval with 2000 bootstrap samples [42]. We followed the suggestions of Preacher and Hayes [39] and calculated the confidence interval of the lower and upper bounds to test whether the indirect, direct, and total effects were significant (Table 3). The magnitudes of the indirect relationship of risk perception, self-efficacy, response-efficacy, and obstacles through behavioral intention to actual disaster preparedness behaviors were 0.036, 0.090, 0.0134, and −0.065, respectively. Because zero is not contained in the CI interval, it can be assumed that behavioral intention is a mediator. The magnitudes of the direct relationship of risk perception, self-efficacy, response-efficacy, and obstacles to actual disaster preparedness behaviors were 0.05, 0.37 (*p* < 0.01), 0.002, and −0.43 (*p* < 0.01), respectively. The total effects of risk perception, self-efficacy, response-efficacy, and obstacles on actual disaster preparedness behaviors were 0.087, 0.461 (*p* < 0.001), 0.136, and −0.492 (*p* < 0.001), respectively. The relationship between risk perception and response-efficacy was fully mediated by behavioral intention. Further, self-efficacy and obstacles were partially mediated by behavioral intention.

## 4. Discussion

This is the first empirical study of cognition behavior to offer clear evidence pertinent to natural disasters in Taiwan. First, the study found that participants’ demographic factors did not have a major influence on actual disaster preparedness behaviors, which means that Hypothesis 1 is not supported. Most of the participants in this study lived near the densely populated earthquake-stricken area, and it was difficult to identify statistical differences that could be used to predict actual behavior. Our study found that women felt more at risk of experiencing earthquakes and had greater behavioral intentions for undertaking disaster preparedness than men. This may indicate that women are more concerned about the safety of their living environment than men. Women are more sensitive to risk and tend to perceive risk more than men [43]. Women are generally better at acknowledging seismic risk because they are more sensitive to their environment [44]. Therefore, future research could explore the importance of increasing men’s perceptions of risk. In addition, future research could emphasize women’s existing risk awareness to strengthen their motivation to undertake disaster preparedness.

We also found that participants with college degrees or higher encountered fewer obstacles to disaster preparedness than those who had not attended college. It is believed that people with higher educational levels are better able to promote disaster preparedness because higher education enhances individuals’ cognitive and learning skills and also provides them access to information and additional resources needed to engage in disaster preparedness [45,46]. Therefore, it is recommended that community education offering disaster preparedness drills or workshops focused on disaster preparedness strategies be provided for people with little education to help them undertake sustainable prevention measures and enhance their preparedness for disasters. The content of education and training can refer to the experience of the United States and Europe [44,47,48]. Russo et al. systematically collated these international experiences and published literature reviews [49,50,51]. They indicated that there are many types of education and training activities (including seminars, workshops, drills, functional exercises, etc.) and determined the quantity of risk reduction that can be obtained from each training activity. They suggested that the content of education and training activities should gradually transition from discussion-based to operation-based exercises, to allow the general public to increase risk perception before learning to increase risk reduction. However, because of the cultural differences between Eastern and Western countries, the key characteristics of, and differences between, the disaster prevention needs of countries cannot be ignored. Therefore, even though education for disaster preparedness is very important, it is necessary to recognize which factors will mainly affect disaster preparedness actions before providing intervention measures.

Second, we turn to the results relevant to Hypothesis 2. Bubeck, Botzen, and Aerts [13] conceptualized disaster preparedness as a social-cognitive process that engenders a self-protective response to a specific threat, which can contribute to an understanding of hazard preparation decisions [52]. Consistent with findings in previous studies, this study’s participants were perceived to have a high level of self-efficacy and high intentions to take preparatory actions [11,53,54,55]. Becker et al. [56] suggest that people with high self-efficacy feel that they have the ability to prevent being harmed, and, as a result of their own efforts and preparations, they will be self-sufficient should they encounter a hazard. It is important to increase peoples’ preparedness abilities and their beliefs that they can do something to mitigate the effects of a disaster by focusing on their emergency preparedness self-efficacy. Becker, Paton, and Johnston [57] and Paton and Johnston [58] provide practical information about how to make these preparations and why they are effective. They suggest beginning with easy-to-adopt items (e.g., emergency kits) and progressively introducing more complex/expensive items (e.g., structural changes to houses) [56].

This study, conducted half a year after the earthquake, found that risk perception did not have a direct effect on actual disaster preparedness behaviors; however, it may be possible that risk perception decreases over time [52]. How people perceive risk will, however, influence their personal behavioral intentions with regard to disaster preparedness [29]. Hence, simply being aware of a risk does not increase the undertaking of actual protective behaviors [28,59,60]. This finding also supports Lindell and Hwang’s [61] observation that the perception of risk might be a necessary step toward preparedness. Our findings showed that both self-efficacy and behavioral intentions were positively associated with actual disaster preparedness behaviors. Obstacles were negatively associated with disaster preparedness behavioral intentions and actual behaviors. The intention to take precautionary actions can be interrupted by obstacles—such as a lack of knowledge, individual skills, time, and finances [11,26,62]—which limit self-efficacy’s capacity to stimulate people to undertake appropriate disaster preparedness [26]. It is possible that when people take protective measures, they form an intention before taking action, which is called “protection motivation”. Further, people may not prepare for disasters because barriers can inhibit the implementation of the decision to take protective actions [59] thereby preventing people from turning their intentions into reality [62]. In other words, motivation to protect themselves was constrained by obstacles they encountered when trying to change their actual behavior. Public health efforts aimed at improving preparedness must focus on the need for a plan [22]. This finding is important, as many public education schemes focus on risk perception rather than on improving self-efficacy and reducing the obstacles to undertaking disaster preparedness. Healthcare providers should recognize the need to address self-efficacy and obstacles to disaster preparedness.

Most participants in this study have a higher intention to prepare for a disaster event than actual behavior. After experiencing the impacts of the 2016 seismic disaster, they have a greatly improved awareness of risk, which directly affects their intentions, however, they have not yet taken actual steps toward disaster preparedness. We suggest that this contradiction is due to the fact that participants were living in the vicinity of the disaster area at that time. Participants’ perceived risk did not directly affect actual disaster preparedness behavior. Thus, Hypothesis 2 was supported in part and not supported in part.

Finally, to address Hypothesis 3, our results showed that behavioral intentions related to risk perception, self-efficacy, response-efficacy, obstacles, and actual disaster preparedness behaviors are an important mediator variable and indicator of actual behavior, and this is especially true with regard to the intention to complete a mediational role in the relationships between risk perception, response-efficacy, and actual behavior. The above results further suggest that risk perception, self-efficacy, and positive response-efficacy encourage people to have stronger intentions that are transformed into actions [10]. Thus, we recommend that these three factors be prioritized to strengthen residents’ actual disaster preparedness behaviors. Research on natural hazards has found that people’s expectations regarding the efficacy of preparedness measures influence their actions. A perceived response-efficacy does not appear to significantly influence actual behaviors of disaster preparedness, but it is a significant factor in influencing participants’ intentions to implement disaster preparedness and is consistent with a previous study’s findings [29]. Because earthquakes are unpredictable, preventive actions cannot guarantee that people will survive when a building collapses. Even if people think they have the ability to prepare emergency kits and emergency plans, their disaster preparedness will not actually achieve the desired outcomes in a critical situation [56,58,63]. Although this study found that response-efficacy does not directly affect actual behaviors, it may directly affect participants’ behavioral intentions.

This study has several limitations. First, the findings cannot be generalized due to the recruitment of a sample of convenience. Second, participants were recruited from sites frequented by ambulatory individuals who were able to carry out most activities of daily living independently, and thus homebound, frail, and disabled individuals were underrepresented. Lastly, this study focused on an earthquake that struck without warning. Other natural disasters, such as floods and hurricanes, can be predicted, and thus findings related to preparations for earthquake disasters cannot be generalized to other types of natural disasters.

## 5. Conclusions

Disaster preparedness cannot control or suppress the occurrence of natural disasters. Nonetheless, it is vital for protecting peoples’ lives, health, and homes, and understanding which factors motivate people to take protective actions contributes to more effective preparedness. Increasing individuals’ capacities—rather than focusing on risk severity and response-efficacy—will lead pre-contemplators to develop stronger intentions to undertake disaster preparedness behaviors. If people are already predisposed to perceive greater levels of self-efficacy and lower barriers or obstacles, then it is important to be able to translate that increased level of motivation into greater preparedness. Our findings can serve as a reference for family and community coping strategies to mitigate damage caused by disasters, despite the inevitability of casualties and losses precipitated by earthquakes. Disaster risk indicators vary by region, and disaster preparedness varies by population. Policy makers may wish to improve family preparations, especially for certain under-prepared social groups. Policy makers should also encourage frequent emergency drills and aim to increase the number of participants in such drills. Our results can be used as a reference for more efficient allocation of limited resources, especially in areas with high risk of large-scale natural disasters and low levels of preparedness.

## Figures and Tables

**Table 1:** Participants’ demographic characteristics (*n* = 286).

Variables		Number (%)
Mean age	38.1 (±10.95) Range 20–79 years	*n* = 268
Gender (F)		187 (65.6)
Education	College or higher	217 (75.9)
Marital status	Married	141 (49.3)
	Single	138 (48.3)
	Divorced	7 (2.4)
Living with families	>65 years	79 (27.6)
	<18 years	123 (43.0)
	Both >65 years and <18 year	46 (16.1)
Religion (Yes)		232 (81.1)
Job (Yes)		252 (88.1)
House	Owner	214 (74.8)
	Rent	57 (19.9)
	Other	15 (5.2)
Fault zone	Yes	34 (11.9)
	No	105 (36.7)
	Unknown	147 (51.4)
Soil liquefaction	Yes	25 (8.7)
	No	124 (43.4)
	Unknown	137 (47.9)

**Table 2:** Means, standard deviations, and correlations of variables (*n* = 286).

	Variables	Items Mean (SD)	1	2	3	4	5
1	Risk perception	3.2 (0.66)	-				
2	Self-efficacy	3.4 (0.71)	0.017	-			
3	Response-efficacy	3.6 (0.69)	0.057	0.586 *	-		
4	Obstacle	2.7 (0.70)	0.172 *	−0.245 *	−0.310 *	-	
5	Behavioral intention	3.5 (0.62)	0.224 *	0.595 *	0.594 *	−0.365 *	-
6	Actual disaster preparedness behaviors	2.6 (0.88)	0.044	0.468 *	0.417 *	−0.323 *	0.534 *

* *p* < 0.01.

**Table 3:** Unstandardized, direct, and indirect effects of the hypothesized model.

Path	Point Estimate	Product of Coefficient		Bootstrapping 2000 Times CI			
				Bias-Corrected		Percentile	
		SE	z	Lower	Upper	Lower	Upper
		Total effect					
RP → ADPB	0.087	0.075	1.160	−0.051	0.244	−0.059	0.231
SE → ADPB	0.461 ***	0.133	3.466	0.204	0.728	0.226	0.746
RE → ADPB	0.136	0.163	0.834	−0.194	0.439	−0.203	0.433
Obstacles → ADPB	−0.492 ***	0.149	−3.302	−0.816	−0.221	−0.803	−0.214
		Directed effect					
RP → ADPB	0.051	0.075	0.680	−0.096	0.204	−0.097	0.203
SE → ADPB	0.371 **	0.136	2.728	0.113	0.639	0.138	0.662
RE → ADPB	0.002	0.178	0.011	−0.338	0.355	−0.379	0.326
Obstacles → ADPB	−0.427 **	0.150	−2.847	−0.785	−0.154	−0.748	−0.149
		Indirect effect					
RP → BI → ADPB	0.036 *	0.023	1.565	0.005	0.100	0.001	0.089
SE → BI → ADPB	0.090 *	0.047	1.915	0.020	0.215	0.009	0.197
RE→BI → ADPB	0.134 *	0.077	1.740	0.015	0.320	0.012	0.315
Obstacles → BI → ADPB	−0.065 *	0.043	−1.512	−0.183	−0.005	−0.170	−0.001

Note: Unstandardized estimating of 2000 bootstrap sample, * *p* ≤ 0.05. ** *p* ≤ 0.01. *** *p* ≤ 0.001.
